# Low-level brain somatic mutations in exonic regions are collectively implicated in autism with germline mutations in autism risk genes

**DOI:** 10.1038/s12276-024-01284-1

**Published:** 2024-08-01

**Authors:** Il Bin Kim, Myeong-Heui Kim, Saehoon Jung, Woo Kyeong Kim, Junehawk Lee, Young Seok Ju, Maree J. Webster, Sanghyeon Kim, Ja Hye Kim, Hyun Jung Kim, Junho Kim, Sangwoo Kim, Jeong Ho Lee

**Affiliations:** 1 Department of Psychiatry, CHA Gangnam Medical Center, CHA University School of Medicine, Seoul, 06135 Republic of Korea; 2 Graduate School of Medical Science and Engineering, Korea Advanced Institute of Science and Technology, Daejeon, 34141 Republic of Korea; 3 Center for Supercomputing Applications, Division of National Supercomputing, Korea Institute of Science and Technology Information, Daejeon, 34141 Republic of Korea; 4 Stanley Medical Research Institute, Laboratory of Brain Research, 9800 Medical Center Drive, Suite C-050, Rockville, MD 20850 USA; 5 Department of Pediatrics, Asan Medical Center Children’s Hospital, University of Ulsan College of Medicine, Seoul, 05505 Republic of Korea; 6 Department of Anatomy, Korea University College of Medicine, Seoul, 02841 Republic of Korea; 7 Department of Biological Sciences, Sungkyunkwan University, Suwon, 16419 Republic of Korea; 8 Department of Biomedical Systems Informatics and Brain Korea 21 PLUS for Medical Science, Yonsei University College of Medicine, Seoul, 03722 Republic of Korea; 9 SoVarGen, SoVarGen, Inc., Daejeon, 34141 Republic of Korea

**Keywords:** Genetics of the nervous system, Genome informatics, Autism spectrum disorders

## Abstract

Low-level somatic mutations in the human brain are implicated in various neurological disorders. The contribution of low-level brain somatic mutations to autism spectrum disorder (ASD), however, remains poorly understood. Here, we performed high-depth exome sequencing with an average read depth of 559.3x in 181 cortical, cerebellar, and peripheral tissue samples to identify brain somatic single nucleotide variants (SNVs) in 24 ASD subjects and 31 controls. We detected ~2.4 brain somatic SNVs per exome per single brain region, with a variant allele frequency (VAF) as low as 0.3%. The mutational profiles, including the number, signature, and type, were not significantly different between the ASD patients and controls. Intriguingly, when considering genes with low-level brain somatic SNVs and ASD risk genes with damaging germline SNVs together, the merged set of genes carrying either somatic or germline SNVs in ASD patients was significantly involved in ASD-associated pathophysiology, including dendrite spine morphogenesis (*p* = 0.025), mental retardation (*p* = 0.012), and intrauterine growth retardation (*p* = 0.012). Additionally, the merged gene set showed ASD-associated spatiotemporal expression in the early and mid-fetal cortex, striatum, and thalamus (all *p* < 0.05). Patients with damaging mutations in the merged gene set had a greater ASD risk than did controls (odds ratio = 3.92, *p* = 0.025, 95% confidence interval = 1.12–14.79). The findings of this study suggest that brain somatic SNVs and germline SNVs may collectively contribute to ASD-associated pathophysiology.

## Brain somatic mutations in autism: insights from high-depth sequencing

Autism Spectrum Disorder is a complex condition influenced by various genetic factors, including inherited traits and new changes in genes. This study investigates the role of low-level brain somatic mutations in ASD. The researchers analyzed brain tissues from deceased individuals, both with and without ASD, using high-depth whole-exome sequencing. The results showed that low-level brain somatic mutations, along with inherited genetic variations, contribute to ASD’s genetic makeup. These mutations were found in genes linked to brain development and function. The study emphasizes the need to consider both inherited and somatic mutations to understand ASD’s genetic complexity. Researchers conclude that the interaction between somatic and inherited mutations is crucial in ASD, providing new insights into its genetic basis. This study enhances our understanding of ASD’s genetic diversity and suggests a multifaceted genetic contribution to the disorder. This summary was initially drafted using artificial intelligence, then revised and fact-checked by the author.

## Introduction

ASD is characterized by phenotypic diversity ranging from defects in social interaction and communication to restricted and repetitive behaviors, interests, or activities. ASD is a genetically heterogeneous disorder^1–3^, with contributions from de novo as well as inherited mutations. Although the heritability of ASD is estimated to be up to 50–90%^4–6^, the genetic accountability for ASD is at most 30%^7–9^. De novo and inherited germline mutations are considered to contribute to ASD^7,10–12^. However, the genetic etiology causing a substantial proportion of sporadic ASD cases remains unclear.

Somatic mutations are postzygotic variations in somatic cells present in a single tissue or several tissues in an organism^13^. Somatic mutations in brain tissues have increasingly been found to be genetic causes of neurological disorders of previously unknown etiology^13,14^. Recent studies have reported that low-level brain somatic mutations, with a less than 5% variant allele frequency (VAF) that arise from neural stem cell niches during brain development or with aging, contribute to various neurological disorders, such as focal epilepsy^15–17^, schizophrenia^18^, and Alzheimer’s disease^19^. Regarding ASD, several studies using brain tissues have sequenced a limited set of known ASD genes and reported the presence of brain somatic mutations in a few ASD-related genes^20,21^. Additionally, recent studies using whole-genome sequencing, of which the read depth ranged from 200x to 360x and was much lower than that of high-depth whole-exome sequencing (>500x), showed that high-level somatic mutations with >5% VAF in neural enhancer sequences or large mosaic copy number variations are associated with ASD^22,23^. These studies support the hypothesis that brain somatic mutations can be implicated in the genetic architecture underlying ASD. However, there is a lack of studies examining whether low-level brain somatic mutations (e.g., less than 5% in VAF) in protein-coding genes are implicated in ASD at the genome-wide level.

Here, we performed high-depth whole-exome sequencing (WES) with an average throughput read-depth of 559.3x to accurately detect brain somatic single nucleotide variants (SNVs) in 181 postmortem tissues. The postmortem tissues comprised 86 specimens of multiple brain regions and 13 specimens of peripheral tissue from 24 ASD subjects, as well as 51 brain and 31 peripheral tissue specimens from 31 neurotypical controls. We performed independent, ultradeep targeted amplicon sequencing (TASeq) to accurately detect low-level brain somatic SNVs. Considering both somatic and germline mutations, we found that low-level brain somatic SNVs are collectively implicated in the pathogenesis of ASD with germline SNVs in ASD risk genes, suggesting that the heterogeneous genetic architecture of ASD may be further explained jointly by somatic as well as germline mutations.

## Materials and Methods

### Tissue collection

Fresh frozen human brain tissue (cortex and cerebellum) and paired peripheral tissue (heart and liver) were generously provided by the National Institute of Child Health and Human Development (NICHD). Samples were obtained from 24 clinically diagnosed autism spectrum disorder (ASD)-affected individuals and 5 age-matched unaffected controls. To increase the number of controls, we additionally acquired DNA from the human dorsolateral prefrontal cortex and peripheral tissues (spleen) of 26 neurotypical controls, which were generously provided by the Stanley Medical Research Institute (SMRI). The human brain and peripheral tissues were acquired from the NICHD and the SMRI, both of which confirmed official consent from all subjects. The research performed on deidentified postmortem human tissues was approved by the Institutional Review Board of the Korea Advanced Institute of Science and Technology.

### DNA extraction and high-depth whole-exome sequencing

Genomic DNA (gDNA) was extracted from the brain and paired peripheral tissues using a QIAamp DNA Mini Kit (Qiagen). We prepared exome libraries following the manufacturer’s protocol (Agilent, Human All Exon V4/V5 + UTR 50 Mb Kit) using up to 1 μg of gDNA as an input. Then, we performed paired-end sequencing on an Illumina HiSeq 2000/2500 instrument (average throughput depth of 559.3×) according to the manufacturer’s instructions using exome libraries that passed quality control (QC-passed). We followed the GATK Best Practices (v3.5) workflow to generate analysis-ready bam files from QC-passed Fastq files. The Fastq files were aligned to a reference genome (GRCh38) using BWA-MEM to generate bam files, and PCR duplicates were marked by Picard. Reads adjacent to indels in the bam fi2000/2500 instrument (RealignerTargetCreator and IndelRealigner from the GATK analysis tools. Finally, we performed recalibration of the base quality scores with BaseRecalibrator from the GATK analysis tools for subsequent variant calling. We publicly deposited our raw fastq files generated from tissues from the National Institute of Child Health and Human Development for 24 ASD subjects and 5 controls (SUB6919131) and also the additional 26 controls from the Stanley Medical Research Institute (SUB6847120).

### Somatic SNV calling

Somatic SNVs were independently detected using MuTect2^24^ (v.3.6.0) and RePlow^25^ for single region-based and multiregion-based candidates, respectively. Both methods were applied to analyze 99 brain and 44 peripheral specimens from 44 individuals (paired cases) and 38 brain-only specimens from 11 individuals (unpaired cases). We ran MuTect2 with default options, including fraction_contamination (default = 0.02). We then checked for cross-contamination between samples using an in-house contamination filter and an independent tool, ContEst^26^. From the MuTect2 output from the paired cases, we excluded unreliable calls by applying the following criteria: (i) variant allele frequency (VAF) ≥ 20%, (ii) EBscore ≤5, and (iii) variants with all supporting reads located at either end of the reads. We also excluded unreliable calls via manual inspection with IGViewer (v2.3.94), checking for the following: (a) supporting reads with altered alleles that had no other base changes, if they were not heterozygotic/homozygotic SNPs; (b) an average for the second highest BLAT scores for supporting reads of <900; and (c) more than 50% of supporting reads (at least three reads). For brain-only samples (unpaired cases), a more strict depth of <300 and a VAF of ≥10% were applied. In addition to MuTect2 calling, we independently ran RePlow for both paired and unpaired cases (see below for more details) and applied the same filter conditions to the outputs. All putative SNVs in protein-coding regions were annotated with the Ensembl Variant Effect Predictor for the characterization of mutation subtypes using the following simplified categories: (a) LOF_MIS, defined as missense, stop gained, start lost, stop lost, splice donor, or splice acceptor variants, and (b) SYN, defined as synonymous variants. For pathogenicity scoring of variants, the Phred-scaled CADD score (v1.5, GRCh38 model) was used, and a cutoff of >20 was applied for identifying putatively pathogenic variants.

### Somatic SNV calling powered by RePlow and barcoded sequencing

The reason for applying RePlow was to rescue SNV candidates filtered out from MuTect2 due to low VAFs. RePlow is a specialized tool designed to detect SNV candidates with low VAFs by utilizing information gleaned from replicates of the same sample. We assumed that if a given SNV candidate was consistently observed from multiple brain regions of the same individual, this would indicate a true variant, despite its low VAF. We considered data from multiple brain regions as pseudoreplicates and applied RePlow to recover such variants. To do so, we first generated initial call sets for each brain region using MuTect2. Then, for each possible pair of brain regions from the same individual (e.g., BA17-BA21), we applied RePlow and selected somatic SNVs identified as true mutations in the initial call sets, which we defined as replicate calls. Among the multiple replicate calls from at least two different region pairs (i.e., three different brain regions), not from the matched peripheral tissue, we confirmed genuine calls using independent ultradeep validation with high-depth sequencing with barcode tags (BCDseq) at an average sequencing read depth of 82,924x. For BCDseq calls, the filter conditions were as follows: (1) >4 supporting barcodes, (2) VAF concordance between WES and BCDseq within a 10-fold change and within a difference of 5%, and (3) conditions where (1) and (2) were satisfied for both regions of an identical variant.

### Empirical Bayesian score

We applied EBscore^27^ to our raw variants from MuTect2 and RePlow calling to distinguish true calls from false-positives based on empirical estimation of the error rates of the variants. The EBscore performance was previously analyzed and described by our group elsewhere^19^. We utilized WES data from 21 independent healthy controls as a panel of reference samples with which to estimate the error rate of each variant position, and we set the EBscore cutoff value to 5 to be conservative in detecting accurate calls.

### Targeted amplicon validation sequencing

Primers were designed with the Primer3 algorithm and synthesized by Macrogen (Seoul, Korea). Amplicons were prepared by two-step PCR using Illumina TruSeq adapters. First, PCRs were carried out using 5 ng of initial template gDNA. The 1st amplicons were analyzed on 2% agarose gels, and bands of the expected sizes were isolated and purified using Mega Quick-Spin Kits (iNtRON, Korea). After purification, 50 ng of the 1st amplicon was used as a template for a second PCR, and the products were subsequently purified with the same purification kit. These 2nd amplicons were quantified using the Bioanalyzer 2100 system (Agilent, USA). QC-passed amplicons were then sequenced on a HiSeq 2500 sequencer (Illumina, USA). The generated Fastq files were aligned to the GRCh38 reference genome by BWA-MEM^28^, and the reads at the target sites were filtered for MQ20 and BQ30 with the bam-readcount R package. We checked the number and quality of altered alleles via visualization with IGViewer^29^ (v2.3.94). For the estimation of background error rates, we performed replicate sequencing with previously constructed spike-in samples. If the identified mutations were statistically reliable (*p* < 0.05) compared to the estimated background error rates, we considered them true calls. The background error rates for the amplicon-based platforms were as follows: T > A (VAF = 0.00312), T > C (VAF = 0.00797), T > G (VAF = 0.000758), C > T (VAF = 0.00407), C > G (VAF = 0.000765), and C > A (VAF = 0.00185)^25^.

### Germline SNV calling

We applied GATK HaplotypeCaller^30^ (v.3.8–0) for calling germline SNVs and included reliable variants in our analysis based on filtering conditions of a total depth ≥100 and an allele frequency ≥30%. We applied the filter conditions to calls obtained concurrently for all brain and peripheral specimens of each subject. To select ASD risk genes with rare and putatively damaging protein-altering germline SNVs, we applied additional conditions, including an ExAC allele frequency <0.02% and CADD score >20. The ASD risk genes were selected from the Simons Foundation Autism Research Initiative (SFARI) database^31^ if they were categorized as class 1 to 3.

### Random permutation test

We collected all genes with putatively damaging brain somatic mutations and the known ASD risk genes (SFARI class 1 to 3) with damaging germline mutations from 24 ASD subjects and 31 normal controls, which totaled a set of 60 unique genes in our cohort. Then, we used the reference gene lists of axon guidance (GO:0007411), neuron projection guidance (GO:0097), dendrite spine morphogenesis (GO:0060997), cellular component morphogenesis (GO:0032989), calcium ion transport into cytosol (GO:0010524), intellectual disability (DisGeNET), mental retardation (DisGeNET), and intrauterine growth retardation (HP:0001511), all of which were identified in advance from EnrichR^32^ by analyzing our merged gene set of ASD subjects (*n* = 18 genes; 7 genes with damaging brain somatic SNVs validated via ultradeep targeted amplicon sequencing and 11 genes with damaging germline SNVs categorized as SFARI class 1 to 3). To simulate the ASD merged gene set overlapping each of the reference genes, we selected 18 random genes from the cohort gene set (*n* = 60) with 10,000 permutations (random resampling). From the permutation distribution of gene overlaps with the reference gene sets, we estimated significance by comparing the actual overlap count with an inferred overlap count cutoff of 5%. We repeated the same permutation test for the 13 merged genes with damaging brain somatic and germline SNVs from normal controls.

### Mutation signature analysis

To determine the contribution of mutation signatures, we pooled all brain somatic SNVs from ASD patients and controls. Then, we formatted the pooled SNVs in VCF files and used them as input files for running Mutalisk^33^. The input files were compared with reference signatures generated by the tool using multiple likelihood estimation followed by linear regression. The best model of signature combinations for somatic SNVs was suggested from the tool by considering both cosine similarity and Bayesian information.

### Protein-protein interactions

Protein‒protein interaction (PPI) datasets were downloaded from the Mentha project^34^. The dataset integrates PPI information where each protein is given a reliability score for its interaction with another protein. We summed the reliability scores of interactions for each protein to produce a weighted number of interactions per protein according to a previously published method^35^. We then compared the weighted numbers of interactions between different groups using two-sided Wilcoxon’s rank sum test.

### Spatiotemporal gene expression analysis

The RNA-seq data of developmental gene expression for human brains were downloaded from BrainSpan^36^. The analysis was limited to 10 stages, from early fetal (10–13 weeks postconception) to middle adulthood (up to 45 years), and included expression values (RPKMs) for all 15 brain regions. We generated developmental signatures of gene expression for each of the 150 combination windows from 10 stages and 15 regions according to the overall formulations of a previous method^37^.

First, we calculated two z scores for each gene i in terms of stage *s* and region *r*. Here, e_i_^*sr*^, which is the gene’s expression in a specific stage and region, was compared to the expression distributions across all stages at *r* to obtain z_i_^*s*^ and across all regions at *s* to obtain z_i_^*r*^, respectively:

$${{z}_{i}}^{s}=\frac{{{{\rm{e}}}_{i}}^{{sr}}-{{m}_{i}}^{s}}{1.4826\cdot {{{\rm{MAD}}}_{i}}^{s}}{\rm{and}}\,{{z}_{i}}^{r}=\frac{{{{\rm{e}}}_{i}}^{{sr}}-{{m}_{i}}^{r}}{1.4826\cdot {{{\rm{MAD}}}_{i}}^{r}}$$

where ${{m}_{i}}^{s}$ and ${{m}_{i}}^{r}$ are the median expression levels of gene i across all regions at stage *s* and across all stages at region *r*, respectively, and MAD*i*^*s*^ and MAD*i*^*r*^ are the median absolute deviations (MADs) of the gene expression across all regions at stage *s* and across all stages at region *r*, respectively. These z_i_^*s*^ and z_i_^*r*^ were then combined into a meta-z score:

$${{z}_{i}}^{{sr}}=\frac{{{{\rm{z}}}_{i}}^{s}+{{z}_{i}}^{r}}{\sqrt{2}}$$

Finally, the genes with ${{z}_{i}}^{{sr}}\ge$ 1.5 were utilized as the expression signatures for each of the 150 combination windows of stages and regions.

We then performed enrichment tests of the 150 spatiotemporal expression signatures for the merged genes (genes with damaging brain somatic SNVs validated via ultradeep targeted amplicon sequencing and genes with damaging germline SNVs categorized as SFARI class 1 to 3) in ASD subjects and normal controls. Fisher’s exact test was applied to calculate the significance of overlaps with all 150 expression signatures. *P* values were corrected for multiple tests using the Benjamini–Hochberg procedure.

## Results

### Identification of low-level brain somatic SNVs through deep sequencing

**Fig. 1:**
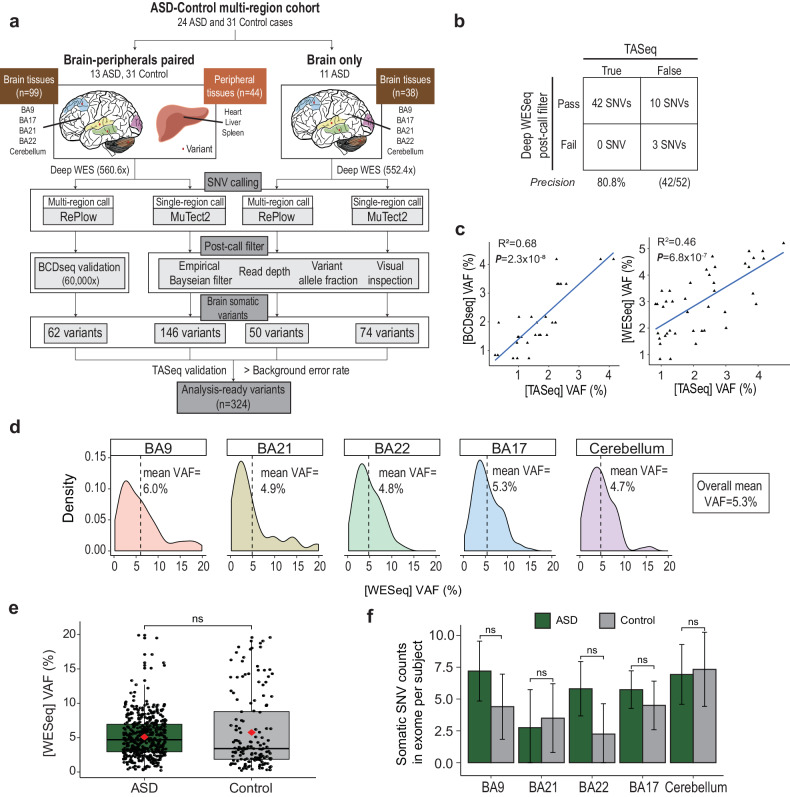
Profiles of brain somatic single nucleotide variants (SNVs). **a** Analytic pipeline. For paired and unpaired brain samples, SNVs were called using different callers and subjected to a unified postcall filter. In total, 324 analysis-ready brain somatic SNVs were identified. **b** Accuracy of the postcall filter. Using 52 random variants, we estimated the precision of the postcall filter to be 80.8%. **c** Concordance in variant allele frequencies (VAFs) between different sequencing platforms. Strong positive correlations were observed between the VAFs of BCDseq and TASeq and between WES and TASeq. **d** Similar VAFs among different brain regions. **e** Similar VAFs between autism spectrum disorder (ASD) patients and controls. Boxplots indicate the median and first and third quartiles; whiskers represent 1.5 times the interquartile range; red diamonds indicate mean VAFs. **f** Similar SNV counts between ASD subjects and controls across different brain regions. Error bars indicate standard errors.

We performed high-depth WES (average throughput read-depth, ASD = 560.6x and control = 552.4x, respectively) on 137 postmortem cortical and cerebellar specimens and 44 peripheral specimens from 24 ASD subjects and 31 neurotypical controls (Supplementary Tables 1 and 2). Among them, 13 of the ASD subjects and all 31 controls had matched peripheral tissues (heart, liver, and spleen) that enabled SNV calling by paired analysis. For the quality control of the raw sequencing data, we confirmed that there was no contamination across the samples using ContEst^26^ and in-house filters (Supplementary Fig. 1a, b). We then established multiple variants calling pipelines optimized for sample conditions: i) paired or unpaired and ii) single or multiple brain regions for each subject (Fig. 1a). Initially, we detected 270 brain somatic SNVs, with an average VAF of 5.8%, in the deep WES data with the application of the somatic mutation caller Mutect2^24^ and filtration strategies, including read depth, VAF cutoff, empirical Bayesian score^27^ and manual inspection. To rescue somatic SNVs with low VAFs under the detection limit of the Mutect2 pipeline, we further applied BCDseq and a replication-aware variant caller (RePlow)^25^ to samples from multiple brain regions to call 62 somatic SNVs with lower VAFs (average 2.7%) (Fig. 1a and Supplementary Fig. 2a). Among them, 54 (87.1%) were undetected in the Mutect2 analysis pipeline. We confirmed that the VAFs of the 54 mutations were concordant among multiple brain regions and also with those from BCDseq, excluding the risk of erroneous calls (Supplementary Fig. 2b). Overall, our comprehensive analysis identified 324 brain somatic SNVs (Supplementary Table 3).

For validation, we randomly selected and subjected 52 of the 324 somatic SNVs (16%) to ultradeep TASeq, which showed that the precision of detecting low-level brain somatic SNVs (average VAF = 2.4%, standard error = 1.84–3.01) was 80.8% (Fig. 1b and Supplementary Table 4). We then compared VAFs between the WES and TASeq datasets and between the BCDseq and TASeq datasets and found that the VAFs of the validated mutations were concordant between the different sequencing platforms (Fig. 1c). The average VAFs were also similar among different brain regions, including BA9, BA21, BA22, BA17, and the cerebellum (6.0%, 4.9%, 4.8%, 5.3%, and 4.7%, respectively) (Fig. 1d), and between ASD patients and control subjects (5.1% and 5.8%, respectively) (Fig. 1e).

The average numbers of brain somatic SNVs per single brain region were 2.3 for ASD subjects and 2.4 for controls (Fig. 1f). For ASD subjects, there was no sex bias in the number of brain somatic SNVs (average number of males = 1.73, average number of females = 2.50; *p* = 0.18, Student’s *t* test). To further investigate sex bias in brain somatic mutations, we collated diverse somatic mutation sources^38^ and found that there was still no female-enriched somatic mutation burden (somatic mutation numbers per brain per individual, males = 3, females = 2.27; *p* = 0.48, Student’s *t* test). We additionally compared the average VAFs of somatic SNVs between paired and unpaired brain samples and found no significant difference between the two (average VAFs, paired samples = 5.3% and unpaired samples = 5.2%, respectively) (Supplementary Fig. 1c). We found no differences among brain regions regarding average throughput read depths (Supplementary Fig. 1d, e). There was no evidence of significant bias according to brain region, paired tissue availability, or disease status. Additionally, regarding VAFs and mutation numbers, there was no significant difference between ASD patients and controls.

For all 55 subjects with an average age of 29.6 years, we estimated the presence of ~2.4 brain somatic SNVs per exome per single brain region, with VAFs as low as 0.3%. By extrapolating this information on a broader genomic scale (~75 million bps to ~3 billion bps), we determined that a total of 96 low-level somatic SNVs per brain may be present at the genome level in ASD patients and controls. Overall, our genetic analyses were able to identify 324 reliable brain somatic SNVs with an average VAF of 5.3% from 24 ASD subjects and 31 neurotypical controls.

### Brain somatic SNVs in ASD patients exhibit comparable mutational loads but are enriched in genes associated with high PPI and neurodevelopment

**Fig. 2:**
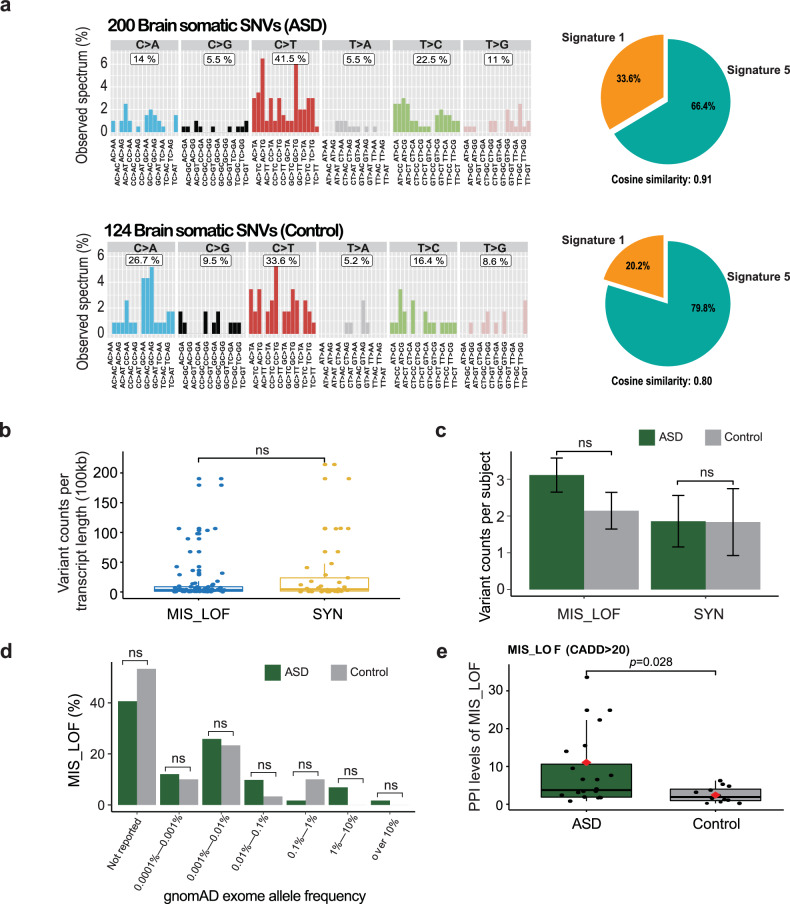
Comparison of functional profiles of brain somatic SNVs between ASD patients and controls. **a** Mutation signature analysis of brain somatic SNVs. Brain somatic SNVs converge on signatures 1 and 5, with cosine similarities of 0.91 and 0.80 in ASD subjects and controls, respectively. **b** Comparison of mutation burden between MIS_LOF and SYN. There was no biased enrichment of transcript length-normalized mutation counts between genes with MIS_LOF and those with SYN. Boxplots depict the median and first and third quartiles; whiskers represent 1.5 times the interquartile range. **c** Numbers of annotated SNVs per subject. The results indicate no difference between ASD subjects and controls for MIS_LOF or SYN. Error bars indicate standard errors. **d** gnomAD exome allele frequency distribution. At least 60% of the subjects were found to carry MIS_LOF, which has not been previously reported in the general population. **e** Protein‒protein interaction (PPI) levels for MIS_LOF. PPI levels for genes with MIS_LOF and CADD scores >20 were greater in ASD patients than in controls. Error bars indicate standard errors.

To examine the potential mechanisms of mutagenesis underlying brain somatic SNVs, we performed mutation signature analysis of the 324 brain somatic SNVs detected in the 55 subjects. We utilized Mutalisk^33^ for the analysis and found that mutation signatures converged on signatures 1 and 5 with cosine similarities of 0.91 and 0.8 in ASD subjects and controls, respectively (Fig. 2a). Mutation signatures 1 and 5 were consistent among subsets of the brain somatic SNVs from different individual mutation callers (Supplementary Fig. 3). Generally, signatures 1 and 5 have been documented in all cancer types and in most cancer samples^39,40^, indicating that a spontaneous, endogenous mutational process may undergird the mutagenesis of the brain somatic SNVs in both ASD subjects and controls in our exome sequencing dataset.

Next, we sought to determine whether the types of brain somatic SNVs differed between ASD patients and controls. To do this, we utilized the Ensembl Variant Effect Predictor (database version 97, GRCh38)^41^. A total of 36.4% of the somatic SNVs (118 out of 324) were annotated within protein-coding regions, among which we specifically chose SNVs presenting as loss-of-function (LOF), missense (MIS), and synonymous (SYN) mutations for further analyses. Between the LOF_MIS and SYN mutations, we examined the mutation burden per gene length^42^, ensuring no biased enrichment in nonsense or nonsynonymous mutations from our exome sequencing dataset (Fig. 2b). We found no significant differences in the number of LOF_MIS or SYN mutations per subject between the ASD patients and controls (Fig. 2c). Interestingly, more than 60% of the LOF_MIS somatic SNVs that we identified had not previously been reported in the genomAD exome allele frequency database^43^ (Fig. 2d). These results indicated that both average mutation counts and population-based minor allele frequencies for somatic SNVs did not significantly differ between ASD patients and controls.

Accordingly, we hypothesized that genes with nonsynonymous mutations would be functionally different between ASD patients and controls. In particular, we focused on genes with putatively damaging mutations, for which we applied Phred-scaled CADD scores^44^ >20 to MIS_LOF (MIS_LOF genes with CADD > 20, ASD = 19 of 51 (37.3%) and control = 12 of 30 (40%)). It was previously reported that highly damaging mutations in ASD-related genes are highly associated with protein‒protein interactions (PPIs)^35^. Generally, the PPI score of a gene indicates the degree of involvement “per gene” in diverse protein‒protein interactions. To obtain the single PPI score per gene, we calculated a weighted sum of the varying PPI scores assigned to a single gene. We then sought to compare PPI levels between ASD patients and controls for the genes with LOF_MIS mutations after classifying genes by high and low CADD scores. The median level of PPIs was greater in ASD subjects than in controls for LOF_MIS mutations with CADD scores >20 (ASD = 4.03 and control=1.93, *p* = 0.024, two-sided Wilcoxon rank sum test) (Fig. 2e). These results showed that damaging brain somatic SNVs in ASD patients are more significantly enriched for genes with high PPIs than in controls, perhaps leading to biological network dysfunction related to ASD.

Given the genetic heterogeneity underlying ASD, various mutational impacts may converge on ASD-related pathogenesis^45^. Thus, we examined whether brain somatic SNVs were found in genes implicated in neurodevelopmental processes or neuropsychiatric disorders. Using ultradeep TASeq, we were able to validate rare damaging brain somatic SNVs in seven different genes with CADD scores >20. Among them, five genes (*ADCY5, CENPJ, DVL1, PEAK1*, and *RGS6*) were not considered high-risk ASD candidate genes in the Simons Foundation Autism Research Initiative (SFARI) dataset^31^ (Table 1 and Supplementary Table 5).

**Table 1:** Subjects carrying damaging brain somatic SNVs.

Subject (n = 6)	Group	Brain somatic SNVs				
		Gene	Mutation	VAF (%)	gnomAD_AF (%)	Neurodevelopmental relevance
4999	ASD	*DVL1*	NM_001330311.2:c.196 C > A (p.D66Y)	0.3	*Not reported*	Social recognition of hierarchy and dominance↓ (PMID: 14960015)
		*ADCY5*	NM_001199642.1:c.2101 G > A (p.H701Y)	1.0	*Not reported*	Dyskinesia↑ (PMID: 26537056)
5144	ASD	*ERBB3*	NM_001982.3:c.1611 T > G (p.N537K)	2.0	*Not reported*	Social novelty preference↓ (PMID: 21547722)
5176	ASD	*PEAK1*	NM_024776.3:c.890 C > T (p.R297Q)	2.5	0.000402	
5403	ASD	*RGS6*	NM_001204424.2:c.335 G > A (p.R112H)	3.0	0.00199	Anxiety and Depression↑ (PMID: 24421401)
5308	ASD	*SLC25A22*	NM_001191060.1:c.718 C > T (p.A240T)	3.8	0.000401	
5841	ASD	*CENPJ*	NM_018451.5:c.3001 G > T (p.Q1001K)	1.7	*Not reported*	Microcephaly (PMID: 15793586)

However, the newly identified genes were associated with defective neurodevelopmental or psychiatric phenotypes. *ADCY5*, known to produce cAMP, which regulates neuronal function, was reported to be related to dyskinesia or Parkinsonian-like motor dysfunction in an *ADCY5*-null mouse model^46^. De novo mutations in the 5’ donor splice site of *ADCY5* were also shown to cause early-onset autosomal dominant chorea and dystonia in a three-generation family study, indicating that *ADCY5* haploinsufficiency is involved in these movement disorders^47^.

A homozygous single-base deletion and missense mutation in *CENPJ* were found to cause microcephaly via centrosome aberrations, and this gene is normally responsible for protein localization to the spindle poles of mitotic cells during prenatal neurogenesis in mice^48^.

*DVL1* was shown to be associated with deficits in the recognition of social hierarchy and dominance in a *DVL1*-null mouse model^49^. Conditional ablation of *ERBB3* in the central nervous system was reported to result in a lack of social novelty preference in a mouse model^50^. *RGS6* knockout in mice was found to inhibit anxiety and depression via serotonin-mediated activation of the 5-HT receptor-adenylyl cyclase axis^51^, suggesting that a gain of function of this gene might be implicated in neuropsychiatric phenotypes^52^.

Notably, three of the aforementioned genes, *ADCY5*, *PEAK1*, and *RGS6*, showed relatively high constraint levels of 0.14 (90% CI 0.08 –0.25), 0.29 (90% CI 0.2 –0.44), and 0.22 (90% CI 0.13 –0.4), respectively, which were calculated by the expected ratios for loss-of-function mutations based on the GenomAD database^43^ (Supplementary Table 6). Furthermore, when using a brain cell-type specificity database based on human single-cell RNA sequencing data^53^, *ADCY5* and *ERBB3* were found to be enriched in oligodendrocytes, while *PEAK1* and *RGS6* were enriched in microglia and neurons, respectively (Supplementary Table 6). Taken together, our results suggested that these genes with rare damaging somatic mutations may be associated with the neurodevelopmental pathogenesis of ASD.

### Damaging somatic and germline SNVs are collectively implicated in the pathogenesis of ASD

Many ASD genetic studies have reproducibly shown that rare damaging germline SNVs in ASD risk genes contribute to the pathogenesis of ASD^54–56^. Thus, from the perspective of the heterogeneous genetic architecture of ASD, we sought to scrutinize whether genes with brain somatic SNVs collaboratively affect ASD-related pathogenesis, with known ASD risk genes carrying rare damaging germline SNVs (CADD score >20, ExAC <0.02%). To do this, we extracted data from subjects with putatively damaging germline SNVs in ASD risk genes among ASD patients and controls. We found that 50% (12 of 24) of the ASD subjects and 25.8% (8 of 31) of the controls had putatively damaging germline SNVs in the ASD risk genes categorized as SFARI ASD risk class 1 (strong), 2 (high), or 3 (suggestive) (Supplementary Table 7). We then merged the newly discovered genes with damaging brain somatic SNVs and known ASD risk genes with damaging germline SNVs in ASD patients to determine whether the merged set of genes converged on biological mechanisms related to ASD pathogenesis. The merged gene set consisted of the genes with damaging brain somatic SNVs validated via ultradeep targeted amplicon sequencing (gene count, ASD = 7 and control = 4, respectively) and the SFARI class 1 – 3 genes with damaging germline SNVs (gene count, ASD = 11 and control = 9, respectively). The merged gene sets thus included 18 genes from 24 ASD subjects and 13 genes from 31 controls. Given that pathogenic mutations are likely located in highly expressed genes involved in tissue development^57^, we hypothesized that the merged gene set with damaging SNVs in ASD patients could overlap with highly expressed genes during fetal brain development, which is associated with the crucial pathobiology of ASD^58^.

**Fig. 3:**
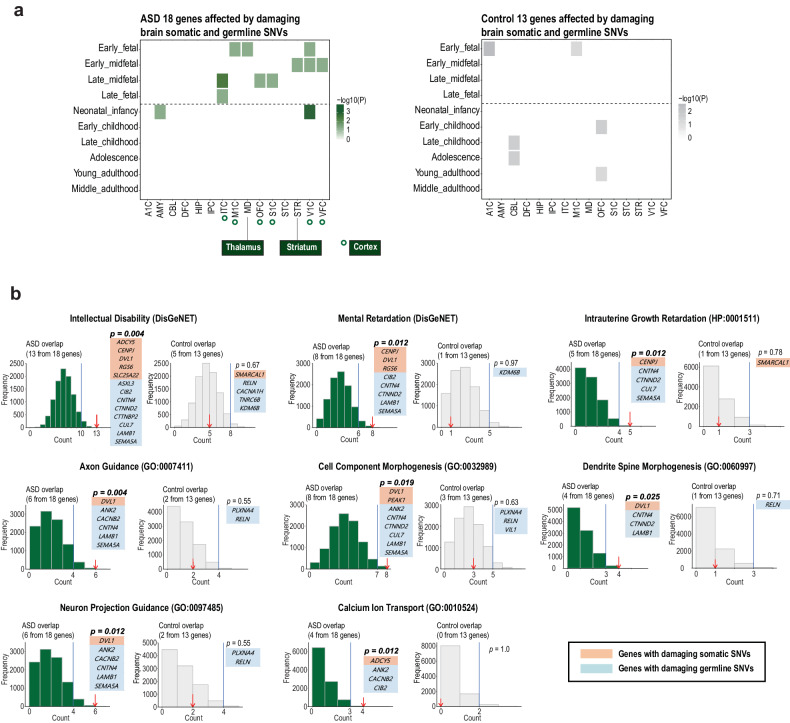
Damaging somatic and germline SNVs are collectively implicated in ASD-related pathobiology. **a** Spatiotemporal gene expression analysis. Spatiotemporal expression analysis was performed for the merged genes (ASD = 18 and control=13) using 150 spatiotemporal gene expression signatures constructed from 15 brain regions and 10 developmental periods. The merged genes consisted of genes with damaging brain somatic SNVs validated via ultradeep targeted amplicon sequencing and genes with damaging germline SNVs categorized as SFARI class 1 – 3. Green and gray boxes indicate significant differences in the number of merged genes overlapping each of the 150 signatures (*p* < 0.05). *P* values were corrected for the Benjamini‒Hochberg procedure. **b** Gene enrichment analysis using random permutation. Gene enrichment analysis was performed for the merged genes (ASD = 18 and control = 13) via random 10,000 permutations. *P* values were adjusted for the false discovery rate using the Benjamini–Hochberg method. The merged genes consisted of genes with damaging brain somatic SNVs validated via ultradeep targeted amplicon sequencing and genes with damaging germline SNVs categorized as SFARI class 1–3. Reference gene lists of biological terms were obtained from DisGeNET^97^, Gene Ontology Biological Process^98^, and Human Phenotype Ontology^99^. Curating the reference gene lists did not involve any presumptions or the use of specific sets associated with brain function-relevant genes. Genes in the orange box and blue box indicate genes with damaging brain somatic SNVs and genes with damaging germline SNVs, respectively. The red arrow and blue vertical line represent the actual and estimated (cutoff=5%) counts of genes overlapping the reference gene lists, respectively.

To test our hypothesis, we constructed 150 spatiotemporal signatures of gene expression from 15 brain regions and 10 developmental periods (Fig. 3a). After calculating how many of the merged gene sets (*n* = 18) overlapped each of the 150 signatures, we found that the genes of ASD patients exhibited significantly high expression levels during the fetal period across ASD-related brain regions, including the cortex^59,60^, thalamus^61^, and striatum^62^ (all *p* < 0.05, Benjamini–Hochberg correction). In controls, however, the merged gene set (*n* = 13) with somatic and germline SNVs did not show significantly enriched gene overlaps for signatures of fetal brains.

To further validate our methodology, we also employed an independent gene expression analysis tool, CSEA^63^, and found similar results: the merged gene set in ASD subjects was enriched in the mid-fetal cortex (*p* = 0.021, Bonferroni correction), but the genes in controls were not in any of the spatiotemporal windows (Supplementary Fig. 4). There were no significant findings from the same analysis when only genes carrying germline mutations were used, probably due to the limited cohort size or the partial genetic accountability for the disease association. Taken together, these results indicated that brain somatic and germline SNVs might impact a collection of genes involved in the aberrant development of fetal brain subregions that are particularly crucial for the pathogenesis of ASD.

With the noted accumulation of damaging brain somatic and germline SNVs, we then analyzed biological terms using EnrichR^32^ and found ASD-related biological terms with adjusted *p* values < 0.05 (Supplementary Table 8). For all of the ASD-related biological terms, we further validated the significance of each of the gene enrichment results by performing 10,000 random permutations, considering a pool of all of the genes with damaging SNVs and the ASD-risk genes with SFARI class 1 –3 from our exome sequencing dataset: axon guidance (GO:0007411) (*p* = 0.004), neuron projection guidance (GO:0097485) (*p* = 0.012), dendrite spine morphogenesis (GO:0060997) (*p* = 0.025), cellular component morphogenesis (GO:0032989) (*p* = 0.019), calcium ion transport into cytosol (GO:0010524) (*p* = 0.012), intellectual disability (*p* = 0.004), mental retardation (*p* = 0.012), and intrauterine growth retardation (HP:0001511) (*p* = 0.012) (Fig. 3b). However, for the merged gene set found in controls, ASD-related or neural development-associated terms were not enriched after the permutation test. The biological process terms for the ASD subjects were reported to be closely related to the pathogenesis of ASD. Briefly, axon pathology, including disruptions in axon growth and projection, has been repeatedly reported in both ASD mouse models^64,65^ and ASD patients^66^. ASD patients exhibit reductions in the size and number of dendrites as well as altered dendrite morphology^67,68^. Aberrations in neuron projections in the human deep cortex during the fetal period have been documented as crucial pathogeneses of ASD^69^. Calcium ion transport activity was reported to be markedly increased in the brain tissues of autistic patients, implicating altered calcium homeostasis in ASD pathology^70^. ASD and other neurodevelopmental conditions, including intellectual disability^71–73^ and mental retardation^74,75^, are thought to have common genetic etiologies, neural circuit alterations, and brain abnormalities such as synaptic transmission. Finally, previous studies indicate that ASD is frequently accompanied by intrauterine growth retardation^76–78^, indicating that the disrupted growth of fetal organs is a critical pathogenesis of ASD.

To further validate the biological implications of the merged gene set in the ASD subjects, we additionally collected ASD-related genes of coexpression modules, which included 10,459 genes from the study of transcriptomic coordination in the developing human prefrontal cortex^79^. Notably, our genes carrying brain somatic and germline mutations still showed enrichment of coexpressed genes (*p* = 0.05, 10,000 random permutations). Overall, these results suggested that brain somatic SNVs may converge with germline SNVs on the pathogenic features observed in ASD.

**Fig. 4:**
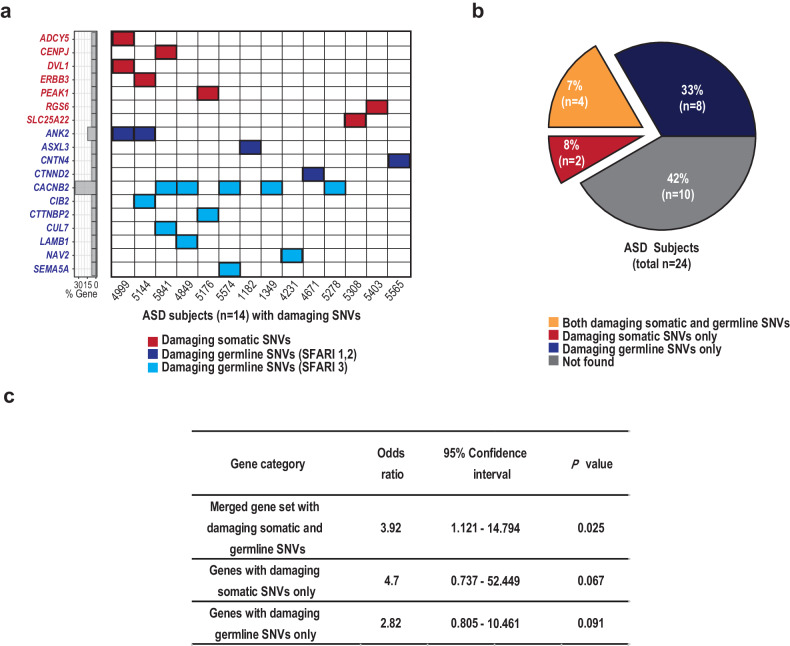
Collective contribution of brain somatic SNVs and germline SNVs to ASD. **a** Heterogeneous genetic architectures of ASD subjects. A total of 58.3% of the ASD subjects (14 of 24) were found to carry genes with damaging brain somatic SNVs, as validated via targeted amplicon sequencing, and/or genes with damaging germline SNVs, categorized as class 1 to 3. Among the 14 patients, 6 carried 2 or 3 genes with damaging SNVs. **b** Proportion of ASD subjects with impactful genes with damaging SNVs. Among the 24 ASD subjects, 14 (58.3%) were found to carry genes with damaging somatic and/or germline SNVs. **c** Assessment of ASD risk was performed by comparing the gene counts of the subsets of ASD subjects (n = 24) and normal controls (n = 31), for the genes with damaging brain somatic SNVs only (6 ASD subjects and 2 controls), the genes with damaging germline SNVs only (12 ASD subjects and 8 controls), and the merged gene set with damaging brain somatic SNVs and damaging germline SNVs (14 ASD subjects and 8 controls). ASD patients carrying the merged gene set with damaging brain somatic and germline SNVs had a significantly greater risk of developing ASD (odds ratio = 3.92, *p* = 0.025, 95% confidence interval = 1.12 –14.79) than did controls.

Finally, we sought to evaluate the combined contribution of brain somatic and germline mutations in ASD patients compared to that in normal controls^80^. To do this, we curated novel genes with brain somatic SNVs, as well as ASD risk genes with damaging germline SNVs, and arrayed the subjects according to gene counts (Fig. 4a and Supplementary Fig. 5a). We found that 58.3% (14 of 24) and 25.8% (8 of 31) of the ASD subjects and controls, respectively, carried at least one gene with either a somatic or germline SNV. Then, we examined whether the collection of brain somatic and germline SNVs was associated with an increased risk of ASD. We compared the number of ASD subjects to that of controls according to the presence of genes with damaging somatic or germline mutations: genes with damaging brain somatic SNVs only (6 ASD subjects and 2 controls), genes with damaging germline SNVs only (12 ASD subjects and 8 controls), and the merged gene sets with damaging brain somatic and germline SNVs (14 ASD subjects and 8 controls) (Fig. 4b and Supplementary Fig. 5b). Interestingly, we found that compared with normal controls, ASD patients carrying the merged gene set with damaging brain somatic or germline SNVs had a significantly greater risk of developing ASD, compared to normal controls (*p* = 0.025, odds ratio = 3.92, 95% confidence interval=1.121 – 14.794, two-sided Fisher’s exact test) (Fig. 4c). However, due to the small size of our cohort (24 ASD individuals and 31 controls), we failed to observe a significant contribution of either genes with damaging brain somatic SNVs only or genes with damaging germline SNVs only to ASD. Taken together, these findings indicated that brain somatic SNVs, in addition to germline SNVs, underlie the heterogeneous genetic architecture of ASD.

## Discussion

In the present study, we generated and exploited a unique resource of deep WES data from 181 postmortem brain and peripheral tissue samples from 24 ASD subjects and 31 neurotypical controls to identify low-level somatic mutations and their contribution to ASD. In doing so, we discovered that brain somatic SNVs in novel genes implicated in neurodevelopmental processes, along with germline SNVs in well-known ASD risk genes, and the merged gene set identified from both somatic and germline mutation sources are associated with the pathogenesis of ASD. The merged gene set affected by either the brain somatic or germline SNVs demonstrated significant enrichment for biological process terms, including axon guidance, neuron projection, and dendrite spine morphogenesis, as well as for other neurodevelopmental condition terms, including intellectual disability, mental retardation, and intrauterine growth retardation. The merged gene set also showed higher gene expression levels during fetal brain development and greater mutation burden in ASD subjects than in normal controls.

In ASD patients, we identified deleterious brain somatic SNVs in *ADCY5*, *CENPJ*, *DVL1, ERBB3*, *PEAK1*, *RGS6*, and *SLC25A22*. Interestingly, five of these genes had previously been found to affect neural commitments: *ADCY5*, which is highly expressed in the striatum, is involved in the modulation of dopaminergic signals and is thus tightly related to motor control^81^. Indeed, in the present study, a subject (ID4999) with a brain somatic SNV in *ADCY5* was reported to suffer from involuntary rhythmic movements. *CENPJ* regulates neural progenitor division and neuronal migration in the cerebral cortex^82^. *DVL1* is required for normal dendritic development in hippocampal neurons^83,84^. *ERBB3* is known to modulate hippocampal neuroplasticity^85,86^. *RGS6* is known to suppress dopaminergic neurodegeneration and motor dysfunction^87^. The other two genes, however, have undergone little study in regard to their neural functions and their relationships with neuropsychiatric conditions. The studies that are available have reported that these genes are closely associated with the pathologic features of ASD. *PEAK1* is known to be a regulator of cell migration^88^, and aberrations in neuronal migration have been documented in individuals with ASD^89,90^. *SLC25A22* constitutes a mitochondrial glutamate carrier^91^, and disruption of mitochondrial carriers by altered calcium signaling has been implicated in ASD pathogenesis^92^.

Notably, the percentage of VAFs of the brain somatic SNVs found in these genes ranged from 0.3 to 3.8%. These low-level somatic mutation burdens in the brain imply that some cells of specific types that are critical for network activity or oscillation, such as inhibitory neurons^93^, or cell nonautonomous mechanisms^94^, may cause defects in the entire brain. How these genes and brain somatic SNVs lead to neural dysfunction or ASD phenotypes remains unclear and warrants further study.

The detection of low-level somatic SNVs generally involves many false-positive calls^95^. To prevent this, we performed high-depth WES at an average throughput read depth of 559.3x. Previous research using the same brain samples only reached an average read depth of up to 95x in target regions and reported negative findings in detecting significant somatic SNVs in ASD brains^21^. A few studies performed only targeted sequencing on confined sets of known ASD risk genes, with no further investigation of novel genes^20^. Additionally, recent studies involving relatively low-depth (~250×) whole-genome sequencing have suggested that somatic mutations in enhancers or mosaic copy number variations may be associated with ASD^22,23^. Although these studies revealed somatic mutations in noncoding regions in ASD patients, low-level somatic mutations in protein-coding regions are generally difficult to detect because of the low read depth of the current WGS approach. Compared with those studies, the high-depth (559.3x) sequencing approach in the present study allowed us to spot low-level somatic mutations with a VAF as low as 0.3%. Furthermore, low-level somatic mutations were found in neurodevelopmental genes that are possibly associated with the pathobiology of ASD (VAF, *DVL1* = 0.3%, *ADCY5* = 1.0%, *CENPJ* = 1.7%, *ERBB3* = 2.0%, *PEAK1* = 2.5%, *RGS6* = 3.0%, and *SLC25A22* = 3.8%). Our results thus support that high-depth sequencing is necessary for the detection of brain somatic SNVs with low-level allele frequencies in novel genes, as well as in known ASD risk genes.

**Fig. 5:**
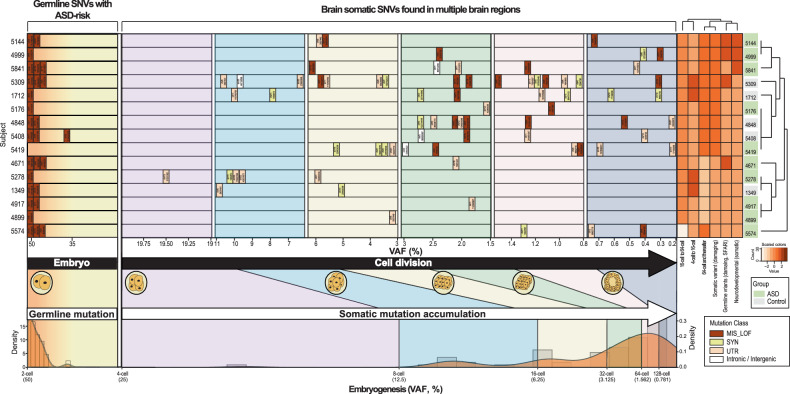
The accumulation of somatic mutations during embryonic cell division. Germline mutations are present from the beginning of embryogenesis. Somatic mutations spontaneously arise in multiple brain regions as embryonic cell division progresses, thereby leading to the accumulation of mutations with low-level variant allele frequencies (VAFs) in the genetic architectures of ASD patients. Prepredisposed germline mutations and accumulating somatic mutations can be simultaneously present in the subjects, thus possibly leading to collective damage to ASD-related neurodevelopmental processes, which is in line with the genetic heterogeneity model of ASD. To differentiate between the ASD patients and controls, unsupervised hierarchical clustering analysis was further performed using features such as the cell division timing of the somatic mutations, the presence of damaging somatic mutations, the presence of damaging germline mutations related to the risk of SFARI ASD, and the neurodevelopmental relevance of the damaging somatic mutations. Some of the ASD subjects (ID 5144, 4999, and 5841; ID 4917 and 4899) were found to be distinctively clustered, whereas the controls were not. For a clear depiction, we used a subset of brain somatic mutations identified from an analysis pipeline using Replow and BCDseq.

By using deep WES followed by strict validation methods, we identified reliable somatic mutations that putatively affect genes key to neural dysfunction related to ASD. To understand the comprehensive genetic architecture of ASD, we also explored rare damaging germline mutations, which are plausibly considered to increase the risk of developing ASD. The approach of combining somatic and germline mutations can in part explain the complex features of the heterogeneous genetic architectures implicated in ASD. Specifically, our results support the idea that the accumulation of brain somatic SNVs during early embryonic or brain development likely contributes to the neural dysregulation and related pathogenesis of ASD in conjunction with predisposed germline SNVs^96^. Both ASD patients and controls were found to have germline mutations in ASD risk genes at their earliest stage of life, embryos (Fig. 5). As embryos undergo cell division, somatic mutations arise and accumulate in genetic architectures that are already predisposed with germline mutations in ASD risk genes. Consistent with this idea, our unsupervised hierarchical clustering analysis, which considered various features, such as the cell division timing of the somatic mutations, the presence of damaging somatic mutations and damaging germline mutations related to SFARI ASD risk, and neurodevelopmental relevance, showed that the ASD subjects (ID 5144, 4999, and 5841; ID 4917 and 4899) were distinctively clustered, whereas the controls were not (Fig. 5). These findings additionally support the hypothesis that somatic and germline mutations can be collectively implicated in the heterogeneous architecture of ASD, although this needs to be replicated in a larger cohort. Nonetheless, the observed biological convergence between somatic and germline SNVs is consistent with an oligogenic model of ASD^96^, wherein diverse mutational sources collectively contribute to ASD risk and its genetic heterogeneity. We cautiously address the oligogenic model-based approach we adopted, which does not mean that mutations occur at the same time but rather that they accumulate and contribute to the overall incidence of ASD. Future studies using a larger ASD cohort with postmortem brain samples will be required to further assess the full contribution of brain somatic mutations to ASD.

## Supplementary information

Supplementary Information

Supplementary Table 1. Sample information summary

Supplementary Table 2. Whole-exome sequencing information of 181 post-mortem brain and peripheral tissues

Supplementary Table 3. High-confidence brain somatic SNVs

Supplementary Table 4. Somatic SNV validation using targeted amplicon sequencing

Supplementary Table 8. Gene enrichment analysis using EnrichR for the merged gene set with damaging brain somatic and germline SNVs in ASD subjects

## Data Availability

The raw high-depth whole-exome sequencing data were deposited in K-BDS (Korea BioData Station) with the accession ID KAP240684.
