# Supplementary material for: Low-level brain somatic mutations in exonic regions are collectively implicated in autism with germline mutations in autism risk genes
Source: Exp Mol Med. 2024 Aug 1;56(8):1750–62. doi: 10.1038/s12276-024-01284-1 (PMC11372092; doi:10.1038/s12276-024-01284-1)
Supplement: Supplementary file 1 — Supplementary Information [file 12276_2024_1284_MOESM1_ESM.pdf]

Supplementary Information

Low-level brain somatic mutations in exonic regions are collectively implicated in autism with germline mutations in autism risk genes

Il Bin Kim[1,2,10], Myeong-Heui Kim[2,10], Saehoon Jung[2], Woo Kyeong Kim[2], Junehawk Lee[3], Young Seok Ju[2], Maree J. Webster[4], Sanghyeon Kim[4], Ja Hye Kim[5], Hyun Jung Kim[6], Junho Kim[7,*], Sangwoo Kim[8,*], Jeong Ho Lee[2,9,11,*]

[1]Department of Psychiatry, CHA Gangnam Medical Center, CHA University School of Medicine, Seoul 06135, Republic of Korea

[2]Graduate School of Medical Science and Engineering, Korea Advanced Institute of Science and Technology, Daejeon 34141, Republic of Korea

[3]Center for Supercomputing Applications, Division of National Supercomputing, Korea Institute of Science and Technology Information, Daejeon 34141, Republic of Korea

[4]Stanley Medical Research Institute, Laboratory of Brain Research, 9800 Medical Center Drive, Suite C-050, Rockville MD 20850, United States

[5]Department of Pediatrics, Asan Medical Center Children's Hospital, University of Ulsan College of Medicine, Seoul 05505, Republic of Korea

[6]Department of Anatomy, Korea University College of Medicine, Seoul 02841, Republic of Korea

[7]Department of Biological Sciences, Sungkyunkwan University, Suwon 16419, Republic of Korea

[8]Deptment of Biomedical Systems Informatics and Brain Korea 21 PLUS for Medical Science, Yonsei University College of Medicine, Seoul 03722, Republic of Korea

[9]SoVarGen, SoVarGen, Inc., Daejeon 34141, Republic of Korea

[10]These authors contributed equally.

[11]Lead contact: jhlee4246@kaist.ac.kr

*Corresponding authors: Junho Kim, Sangwoo Kim, Jeong Ho Lee

Supplementary Figures

Supplementary figure 1

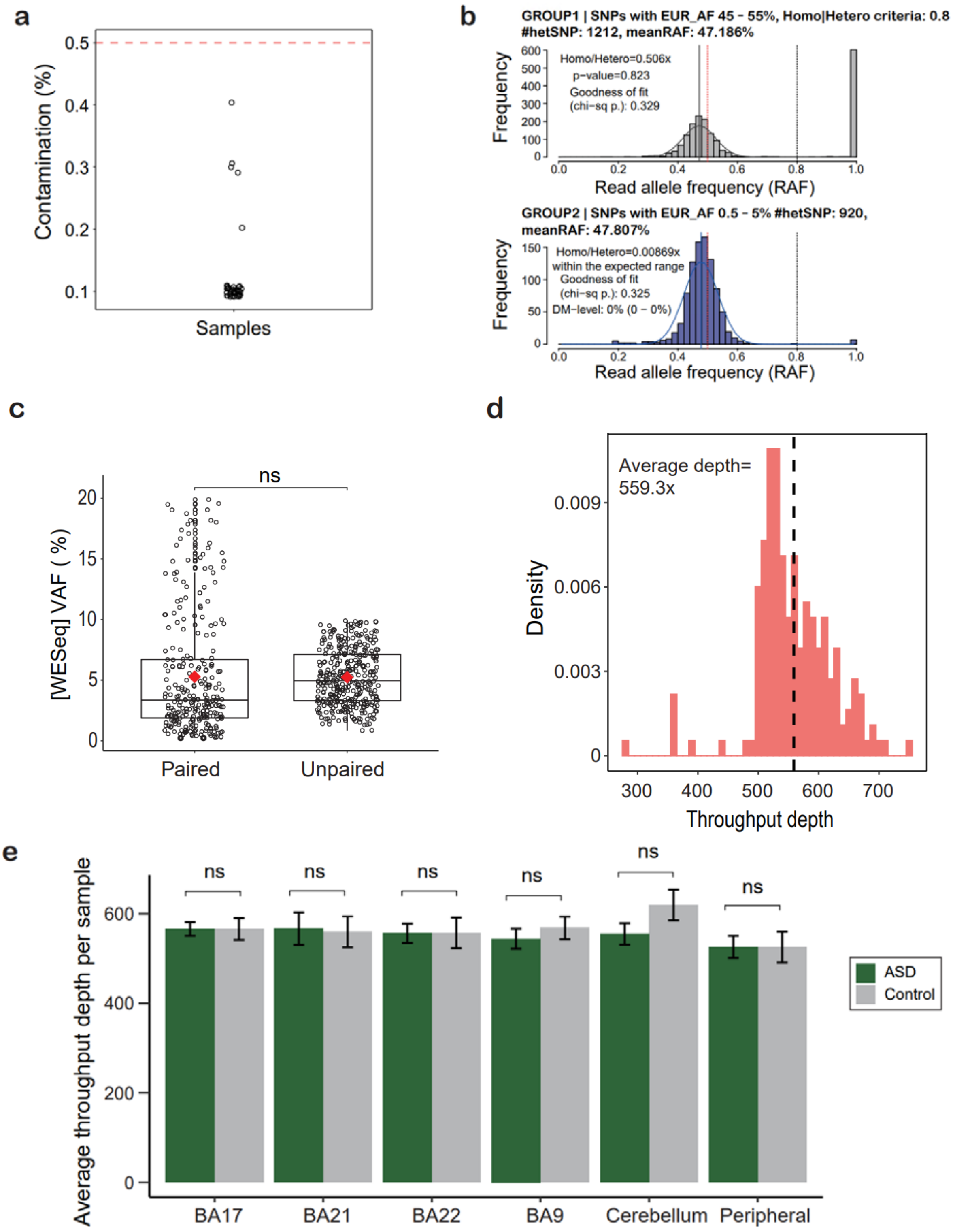

Supplementary Fig. 1. Reliability of analysis-ready bam files. a, Sample contamination was examined using ContEst. All bam files were found to be free of contamination (cut-off portion

<0.5%). **b,** Example of results from running in-house contamination filters. The results show that read allele frequencies (RAFs) in a sample are distributed across single nucleotide polymorphisms (SNPs) in a general population at different allele frequency ranges (upper, 45–55% and lower, 0.5–5%). RAFs in the sample normally build two separate distributions of heterozygous and homozygous SNPs as shown in this plot, indicating no cross-contamination in the sample. **c,** Similar VAFs between paired and unpaired samples. Boxplots indicate the median and first and third quartiles; whiskers represent 1.5 times the interquartile range; red diamonds indicate mean VAFs. **d,** Sequencing depth distribution. Average throughput depth for all samples was 559.3x for 181 post-mortem brains and peripheral tissues. **e,** Average throughput depth comparison between ASD subjects and controls. Throughput depths were similar between the two groups across different brain regions. Error bars indicate standard errors.

Supplemetary figure 2

a

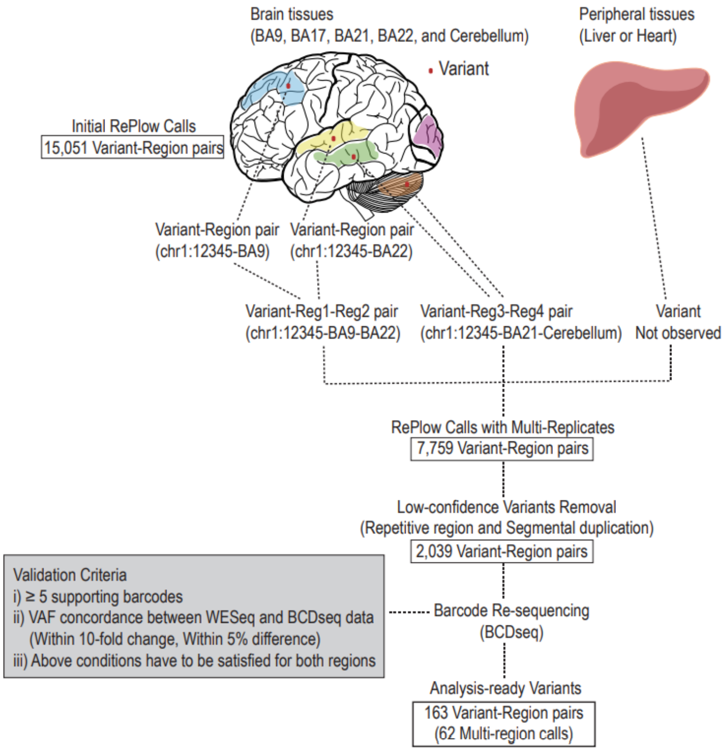

b

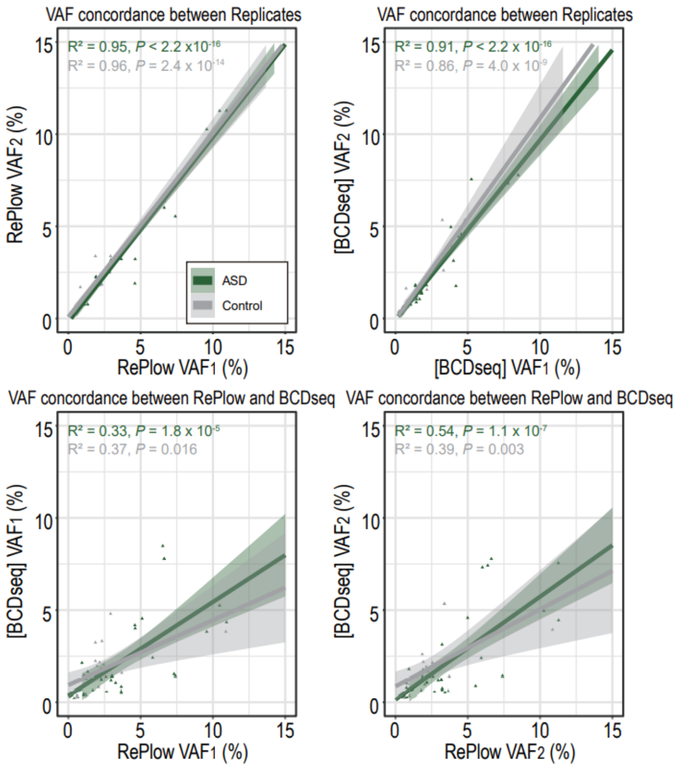

Supplementary Fig. 2. Calling pipeline powered by RePlow and BCDseq. a, RePlow calling and subsequent BCDseq validation algorithm. RePlow was initially applied to extract variant-region pair calls that were consistently called in at least two different brain regions for the same

subject, defined as replicate calls. Among the multi-replicate calls recruited from at least three brain regions, but not from peripheral tissue, we confirmed genuine calls using independent ultra-deep validation BCDseq with in-house validation criteria. **b,** VAF concordance between the different calling algorithms. VAFs were similar among RePlow replicate calls (R^2=0.66–0.96) and among RePlow and BCDseq calls (R^2=0.33–0.54).

Supplementary figure 3

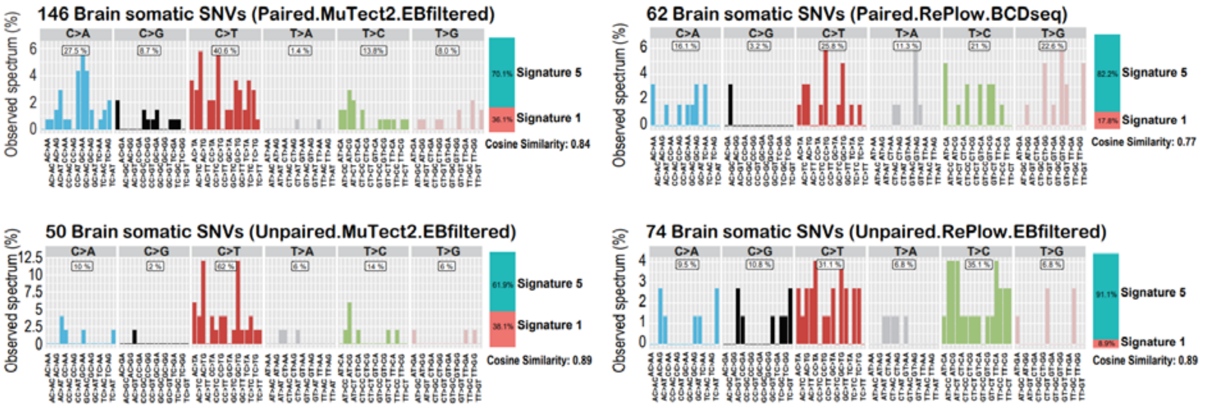

Supplementary Fig. 3. Mutation signature analysis by mutation calling pipelines. Mutation signature analysis consistently identifies signatures 1 and 5 for subsets of brain somatic SNVs extracted from different mutation calling pipelines.

Supplementary figure 4

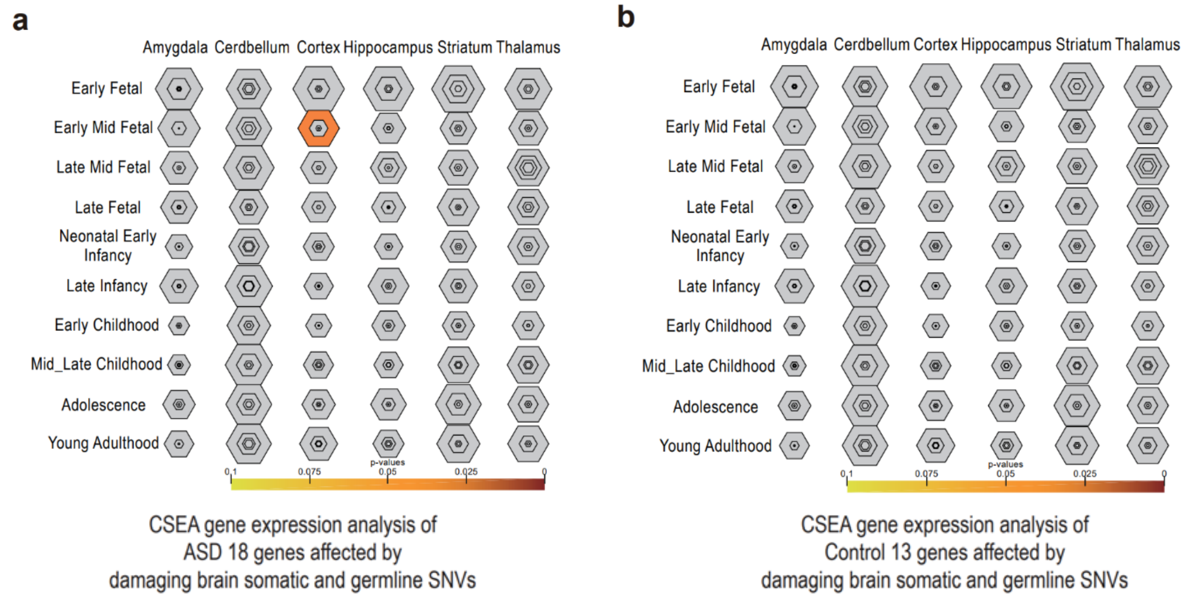

Supplementary Fig. 4. Spatiotemporal gene expression analysis using CSEA. Spatiotemporal gene expression analysis was performed using CSEA for the merged gene sets of ASD subjects (n=18) **(a)** and normal controls (n=13) **(b)**. Early mid-fetal cortex was significantly enriched only for the merged gene set of ASD subjects (*p*=0.021, Bonferroni correction). The merged gene set consisted of the genes with damaging brain somatic SNVs validated via ultra-deep targeted amplicon sequencing and the genes with damaging germline SNVs categorized as SFARI class 1–3. Varying stringencies for enrichment are represented by the size of the hexagons going from least specific lists (outer hexagons) to most specific (center). Hexagons are scaled to size of gene lists.

Supplementary figure 5

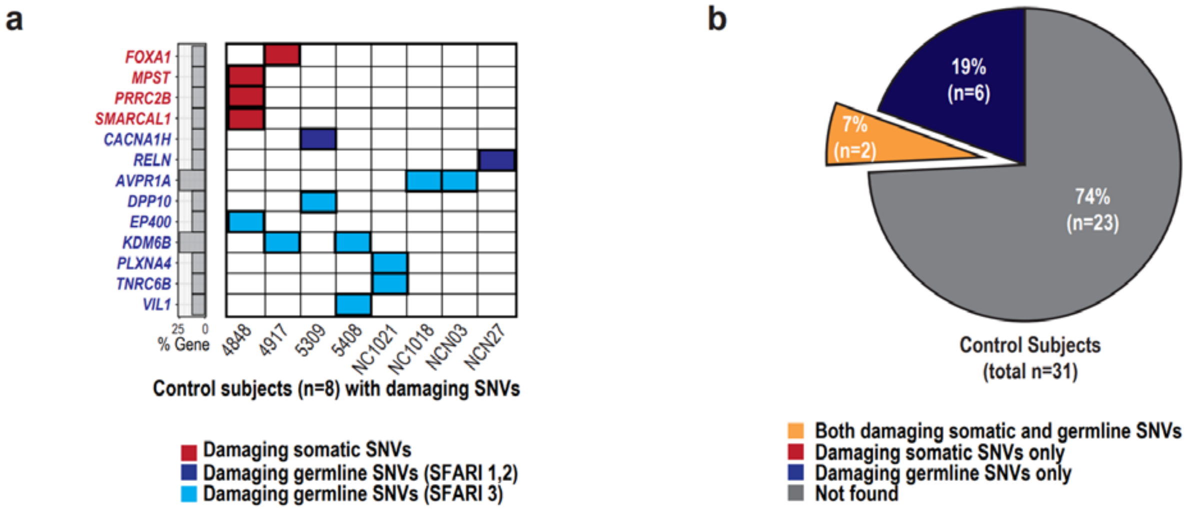

Supplementary Fig. 5. Brain somatic and germline SNVs of normal controls. a, 25.8% of normal controls (8 of 31) were found to carry genes with damaging brain somatic SNVs validated via targeted amplicon sequencing and/or genes with damaging germline SNVs categorized as class 1–3. **b,** Proportion of controls with genes with damaging SNVs. Among 31 controls, 8 (25.8%) were found to carry genes with damaging somatic and/or germline SNVs.

Supplementary Tables

Supplementary Table 1. Sample information summary (provided as an Excel file)

Supplementary Table 2. Whole-exome sequencing information of 181 post-mortem brain and peripheral tissues (provided as an Excel file)

Supplementary Table 3. High-confidence brain somatic SNVs (provided as an Excel file)

Supplementary Table 4. Somatic SNV validation using targeted amplicon sequencing (provided as an Excel file)

Supplementary Table 5. Control subjects carrying damaging brain somatic SNVs

Supplementary Table 6. Constraint ratios and brain cell type specificity for genes with somatic SNVs

Supplementary Table 7. Subjects carrying SFARI high-risk genes with rare damaging germline SNVs (ExAC <0.02 and CADD score >20)

Supplementary Table 8. Gene enrichment analysis using EnrichR for the merged gene set with damaging brain somatic and germline SNVs in ASD subjects (provided as an Excel file)

Supplementary Table 5. Control subjects carrying damaging brain somatic SNVs

Subject (n=2)	Group	Brain somatic SNVs				
		Gene	Mutation	VAF (%)	gnomAD_AF (%)	Neurodevelopmental relevance
4848	Control	*SMARCAL1*	NM_001127207.1:c.1700C>T (p.P567L)	2.6	0.0003986	
		PRRC2B	NM_013318.3:c.6250G>A (p.G2084S)	1.1	0.008245	
		MPST	NM_021126.5:c.413G>T (p.R138L)	1.7	*Not reported*	
4917	Control	*FOXA1*	NM_004496.3:c.G119A (p.S40F)	3.0	*Not reported*	

Supplementary Table 6. Constraint ratios and brain cell type specificity for genes with somatic SNVs

Subject (n=8)	Group	Genes with brain somatic SNVs	Observed/expected[α] (90% CI)	Enriched human brain cell type[ß]
4999	ASD	*DVL1*	0.47 (0.31-0.73)	
		ADCY5	0.14 (0.08-0.25)	Oligodendrocyte
5144	ASD	*ERBB3*	0.52 (0.4-0.69)	Oligodendrocyte
5176	ASD	*PEAK1*	0.29 (0.2-0.44)	Microglia
5403	ASD	*RGS6*	0.22 (0.13-0.4)	Neuron
5308	ASD	*SLC25A22*	0.41 (0.22-0.81)	
5841	ASD	*CENPJ*	0.77 (0.61-0.97)	
4848	Control	*SMARCAL1*	0.41 (0.28-0.61)	
		PRRC2B	0.12 (0.08-0.19)	
		MPST	0.75 (0.44-1.34)	
4917	Control	*FOXA1*	0.26 (0.12-0.68)	

α, gnomAD gene constraint information presented by observed to expected ratios. ß, Cell type enriched for genes based on human single cell RNA sequencing dataset

Supplementary Table 7. Subjects carrying SFARI high-risk genes with rare damaging germline SNVs (ExAC <0.02 and CADD score >20)

Subjects (n=20)	Group	Germline SNVs with SFARI high-risk		
		**Gene**	**Mutation**	**SFARI**
1182	ASD	*ASXL3*	NM_030632.3:c.6359C>T (p.A2120V)	High
1349	ASD	*CACNB2*	NM_000724.3:c.1800T>G (p.D600E)	Suggestive
4231	ASD	*NAV2*	NM_001244963.2:c.1029C>G (p.P57R)	Suggestive
4671	ASD	*CTNND2*	NM_001288715.1:c.3263C>T (p.R1088H)	Strong
4849	ASD	*CACNB2*	NM_000724.3:c.1800T>G (p.D600E)	Suggestive
4849	ASD	*LAMB1*	NM_002291.3:c.2542C>G (p.A848P)	Suggestive
4999	ASD	*ANK2*	NM_001127493.2:c.3002A>C (p.K1001T)	High
5144	ASD	*ANK2*	NM_001127493.2:c.166G>A (p.V56M)	High
5144	ASD	*CIB2*	NM_001271888.1:c.98G>A (p.A33V)	Suggestive
5176	ASD	*CTTNBP2*	NM_001363349.1:c.4801T>G (p.K1601Q)	Suggestive
5278	ASD	*CACNB2*	NM_000724.3:c.1800T>G (p.D600E)	Suggestive
5565	ASD	*CNTN4*	NM_001206955.1:c.662G>A (p.G221D)	Strong
5574	ASD	*CACNB2*	NM_000724.3:c.1800T>G (p.D600E)	Suggestive
5574	ASD	*SEMA5A*	NM_003966.3:c.2272C>A (p.D758Y)	Suggestive
5841	ASD	*CACNB2*	NM_000724.3:c.1800T>G (p.D600E)	Suggestive
5841	ASD	*CUL7*	NM_001168370.1:c.4227C>A (p.G1409H)	Suggestive
4848	Control	*EP400*	NM_015409.5:c.3007G>C (p.D1003H)	Suggestive
4917	Control	*KDM6B*	NM_001080424.2:c.607C>G (p.P203A)	Suggestive
5309	Control	*CACNA1H*	NM_001005407.1:c.3916C>G (p.L1306V)	Strong
5309	Control	*DPP10*	NM_001004360.3:c.990A>C (p.L330F)	Suggestive
5408	Control	*KDM6B*	NM_001080424.2:c.607C>G (p.P203A)	Suggestive
5408	Control	*VIL1*	NM_007127.2:c.128G>A (p.G43D)	Suggestive
NC1018	Control	*AVPR1A*	NM_000706.4:c.924G>T (p.F308L)	Suggestive
NC1021	Control	*PLXNA4*	NM_020911.1:c.5608G>A (p.H1870Y)	Suggestive
NC1021	Control	*TNRC6B*	NM_001024843.1:c.46G>A (p.V16M)	Suggestive
NCN03	Control	*AVPR1A*	NM_000706.4:c.924G>T (p.F308L)	Suggestive
NCN27	Control	*RELN*	NM_005045.4:c.3839C>T (p.G1280E)	Strong
